# Supplementary material for: Nonlinear sensitivity of glacier mass balance to future climate change unveiled by deep learning
Source: Nat Commun. 2022 Jan 20;13:409. doi: 10.1038/s41467-022-28033-0 (PMC8776880; doi:10.1038/s41467-022-28033-0)
Supplement: Supplementary file 1 — Supplementary Information [file 41467_2022_28033_MOESM1_ESM.pdf]

Nonlinear sensitivity of glacier mass balance to future climate change unveiled by deep learning

Jordi Bolibar, Antoine Rabatel, Isabelle Gouttevin, Harry Zekollari and Clovis Galiez

Supplementary Materials

Glacier evolution in the Mont-Blanc and Écrins regions

The Mont-Blanc and Écrins regions, with the latter being composed by the Pelvoux, Oisans and Champsaur massifs, host the highest mountains in the French Alps. From a historical point of view, these two regions (Fig. S4) have played a very important role in the relationships between the French and European societies and mountains[1], being widely acknowledged as the birthplaces of alpinism and mountaineering[2,3]. These mountains, including their glaciers, have shaped the socioeconomic models of these regions, with well-developed tourism and hydropower industries[3,4]. Our results indicate an average projected loss of 58 (-59%) and 49 (-91%) km^2 of glacierized surface in the Mont-Blanc and Écrins regions, respectively, and a total of 185 km^2 in the whole French Alps under RCP 4.5. The landscapes of these two emblematic mountain regions are expected to be greatly transformed (Fig. S4), with only a few high-altitude glaciers capable of coping with the future warmer climate, such as the Bossons (S4c) and Taconnaz (S4d) glaciers around the Mont-Blanc summit (4810 m.a.s.l.) or the Glacier Blanc (S4e) next to the Barre des Écrins (4102 m.a.s.l.). Highly touristic sites like the Mer de Glace (S4b) and Argentière (S4a) glaciers close to Chamonix, and the glaciers around La Meije summit next to La Grave will lose between 45 and 82% of their area under RCP 4.5. The case of Mer de Glace, the largest glacier in the French Alps (29 km^2 in 2015) is quite representative of this trend, with an expected loss of half of its surface area and 65% of its volume by the end of the century, losing the entirety of its emblematic tongue that gave its name (Fig. S4b).

Glacier survival factors in the French Alps

Glacier retreat modulates the interplay between the two main factors that determine glacier MB: climate and topography. A statistical analysis of model results revealed that glacier maximum altitude, latitude and longitude are the most important factors for glacier survival in the French Alps, explaining 69% of the remaining glacierized fraction of glaciers by the end of the century (see Statistical Analysis). A high-altitude accumulation basin is the most decisive factor for a glacier to survive the future warmer climate (53% of importance, $p < 0.01$), ensuring great amounts of solid precipitation and a large cold area to retreat to. In a second term, glaciers in the northern massifs receive increased amounts of precipitation due to the more intense western fluxes and higher latitude (34% of importance, $p = 0.08$; e.g. Chablais and Mont-Blanc in Fig. 2d). The relationship with longitude is not statistically significant (13% of importance, $p = 0.57$), implying a minor role on modulating glacier change compared to latitude, likely explained by the relatively

narrow range of longitudes covered by the French Alps. Despite occupying a relatively small area (Fig. 2b), the French Alps display notorious differences in climatic conditions due to their particular geographical position, with an increasing precipitation gradient spanning from southeast to northwest (Fig. 2d) (*5*). On the one hand, the southern massifs (1-5 in Fig. 2) have a Mediterranean climate influence compared to the northern ranges (6, 8, 10, 11 in Fig. 2), which tend to receive increased precipitation from western Atlantic fluxes. Alternatively, eastern glaciers close to the Italian border (7 and 9 in Fig. 2) receive less precipitation, mainly from east returns. This type of precipitation events can have a very local extent, producing different amounts of accumulation between eastern and western glaciers. On the other hand, topography conditions glacier altitude, which modulates temperature and snowfall on glaciers. Massifs with vast high-altitude accumulation basins (e.g. the Mont-Blanc and Pelvoux massifs), provide areas with colder climates for glaciers to retreat to. Conversely, low-altitude massifs (e.g. Belledonne and Chablais) can no longer sustain glaciers with the present climate despite high amounts of snowfall (Fig. 2d), with their small glaciers being remnants of the Little Ice Age. Such glaciers currently survive thanks to very specific topographical configurations, such as steep north-facing slopes or snow-feeding avalanche couloirs, that help to reduce the high ablation rates typical from these low altitudes. Glaciers in these massifs are projected to disappear within the next two to three decades (Fig. 2).

Mass loss mitigation through glacier geometry adjustment

Another important aspect of climate-glacier forcings is the role of glacier geometry change. Glaciers are excellent climate proxies, fluctuating with climate variations. They advance or retreat in order to reach equilibrium with the climatic conditions[5]. In order to study these climate-glacier interactions, we analyzed the consequences of glacier geometry changes on the climate signal received by glaciers. Its effects on annual CPDDs, snowfall, rainfall and glacier-wide MB (Fig. S8), computed at the glaciers' mean altitude, were quantified by comparing model projections with an evolving glacier geometry against projections with a constant initial geometry. This comparison highlights how glaciers retreating to higher altitudes encounter greatly modified climatic conditions, experiencing reduced temperatures up to 400 PDDs a^{-1} for the highest greenhouse gases concentration scenario and consequently reduced melt (Fig. S8a). Precipitation-wise, glacier retreat induces an important reduction in rainfall (up to -120 mm a^{-1}) and an increase in snowfall (up to 230 mm a^{-1}), helping the glacier transition towards equilibrium (Fig. S8d,g). This change in climatic conditions has important consequences for glacier MB. The reduced melt and increased accumulation limit glacier mass loss, with annual differences up to 1.2 m.w.e. by the end of the century in the region. Despite this significant mitigation of glacier mass loss, our projections indicate that glacier retreat will not be sufficient to allow the glaciers to reach equilibrium with the future climate under any projected climate scenario in the French Alps (Fig. S1a).

The benefits of a nonlinear mass balance model

We showed that by using a nonlinear glacier MB model based on deep learning, important nonlinearities in the response of glaciers to climate forcing are captured. A thorough cross-validation analysis from a previous study indicated that deep learning models provide a more accurate representation of nonlinear glacier mass changes compared to linear models, with improvements up to +108% in explained variance[6]. These nonlinearities are not considered in currently used MB models, whose linear relationships are only accurate for a certain range of MB rates, being specifically fitted for the main cluster of MB values used for training or calibration. As most MB distributions are Gaussian or Gumble-type[7], this calibration is performed around the median values, where the highest concentration of data is found, thus reducing the loss function used for calibration (e.g. the root mean squared error, RMSE). Such a calibration produces a model that is accurate for the majority of MB rates, at the cost of sacrificing performance for extreme values. In the current context of strong glacier retreat, these median MB values are normally negative[8], implying a drop in performance for extremely negative and neutral-to-positive MB rates (Fig. S9). Our analyses suggested that this particular behaviour is likely found in both machine learning (statistical) and temperature-index (empirical) models. A poor representation of extreme values is a core problem in modelling, even for nonlinear models. Nonetheless, this effect was found to be strongly reduced by deep learning models, due to their superior nonlinear explained variance[6]. Our results also serve as a validation of the use of linear MB models for rather homogeneous climate conditions. In the absence of climate extremes, linear models successfully reproduce glacier MB rates, with a reduced bias similar to nonlinear models. However, their accuracy is still systematically lower than deep learning models, thus yielding unbiased but less accurate predictions[6].

Statistical analysis

The statistical analysis on the main factors determining glacier survival in the French Alps was performed via a classic least-squares linear regression with the Statsmodels Python library[53]. A linear regression model was fitted based on the following topographical characteristics of glaciers: the maximum glacier altitude, the average glacier slope throughout the century, and latitude and longitude. These predictors were fitted to predict the ice volume fraction by 2100 for each glacier, computed as the ice volume in 2100 divided by the ice volume in 2015. Results were determined by extracting the standardized coefficients given to each of the predictors, enabling the computation of the importance and contribution of each one of them. P values (t-test) served to determine if predictors were significant or not, providing the degree of trust in the results.

Supplementary figures

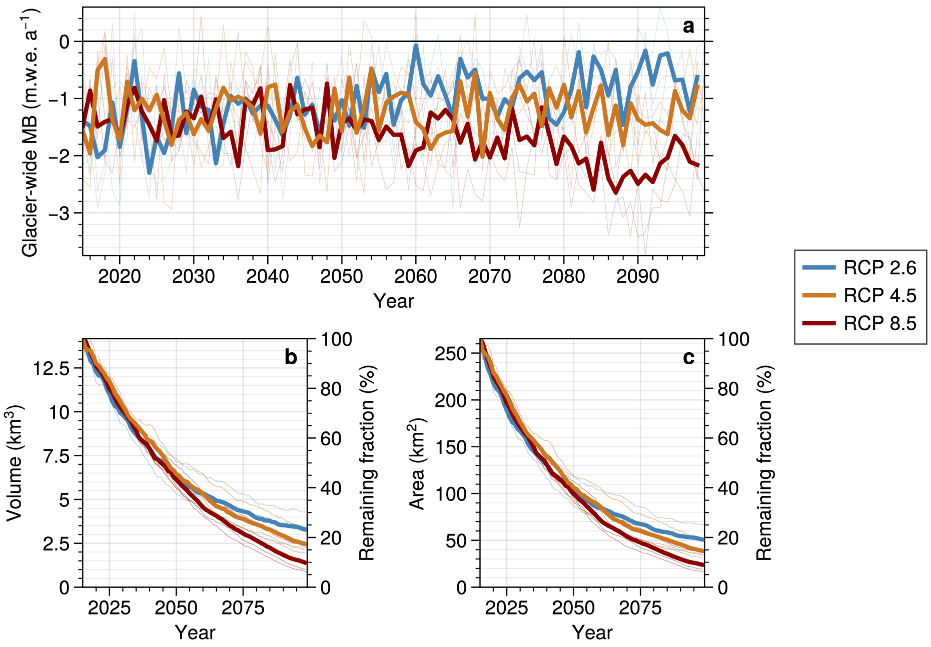

Fig. S1. Glacier-wide MB, volume and area evolution of French Alpine glaciers through the 21st century for climate members including RCP 2.6. Glacier-wide MB (a), ice volume (b) and surface area (c) projections under RCP 2.6, 4.5 and 8.5

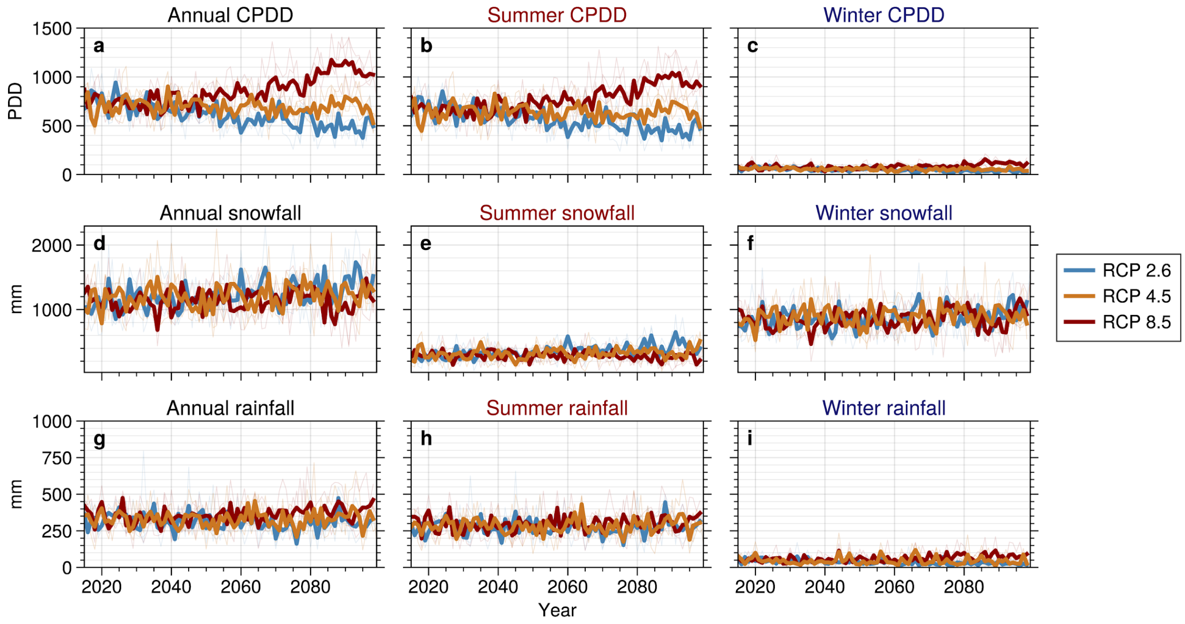

Fig. S2. Climate signal over glaciers in the French Alps for climate members including RCP 2.6. The average climate signal, computed at the glacier's annually evolving centroid, displays the average climate forcing (**a-c**: positive degree-days, **d-f**: snowfall, **g-i**: rainfall) on glaciers

taking into account glacier geometry change. Summer climate is computed between April 1st and September 30th and winter climate between October 1st and March 31st.

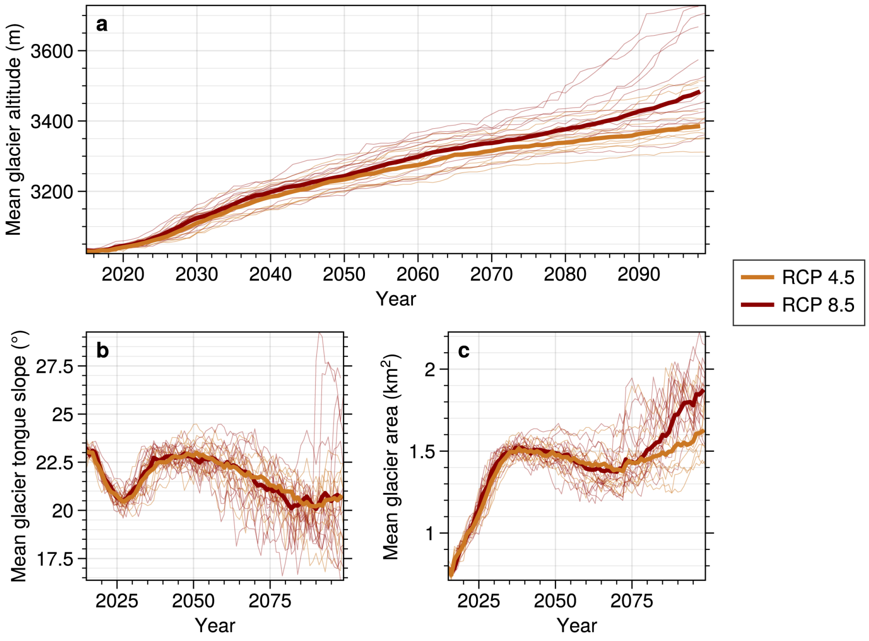

Fig. S3. Projections of glacier topographical characteristics. (**a**) Mean glacier altitude projections, (**b**) Mean slope of the lowermost 20% altitudinal range of glaciers, as a proxy of the glacier's tongue slope, (**c**) Mean glacier surface area projections.

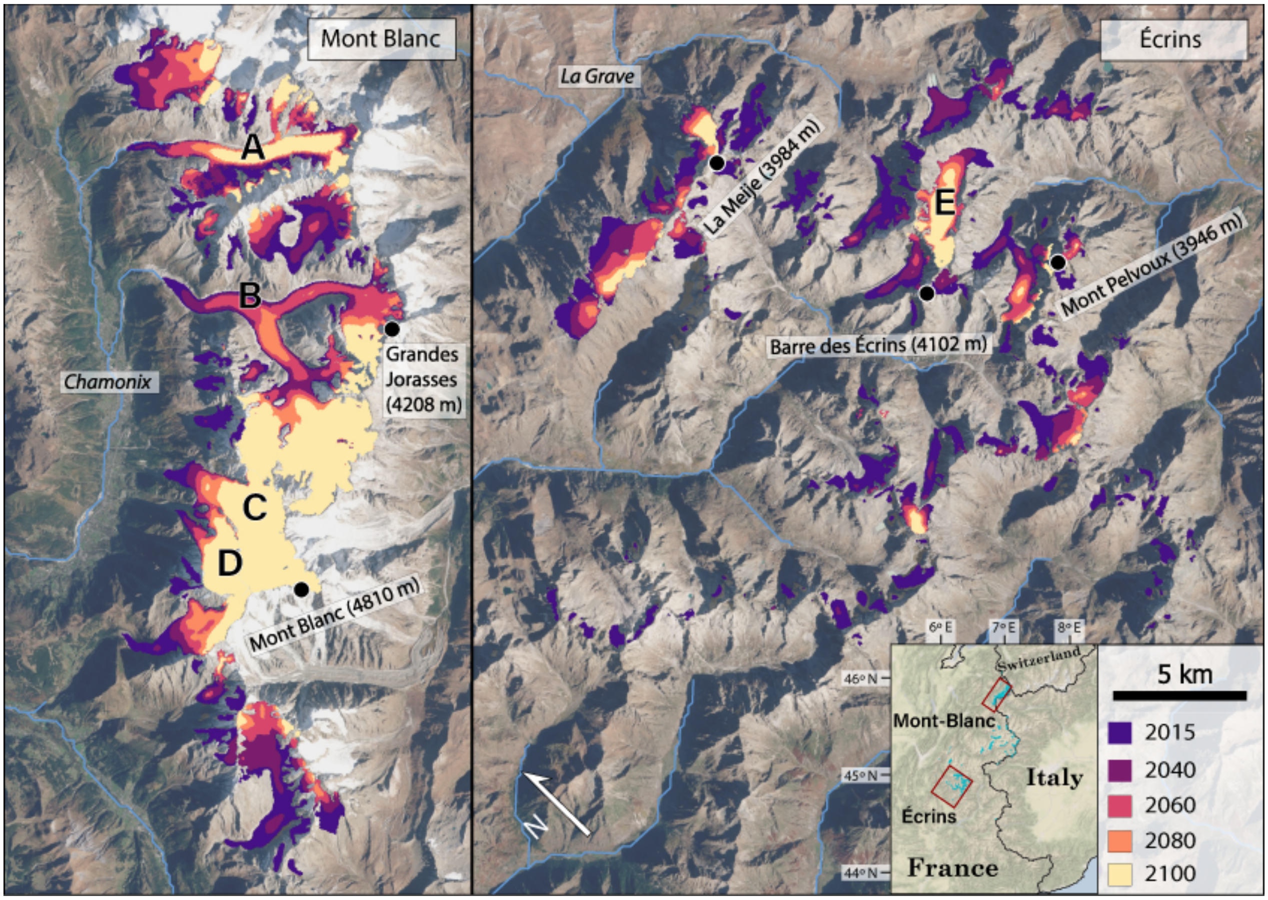

Fig. S4 Projected glacier evolution in the Mont-Blanc and Écrins regions. Projections under CLMcom-CCLM4-8-17_CNRM-CERFACS-CNRM-CM5 RCP4.5, being the closest to the multi-model air temperature and precipitation mean. Specific glaciers (**A)** Mer de Glace, **(B)** Argentière, **(C)** Bossons, **(D)** Taconnaz and **(E)** Glacier Blanc are referenced in the main text.

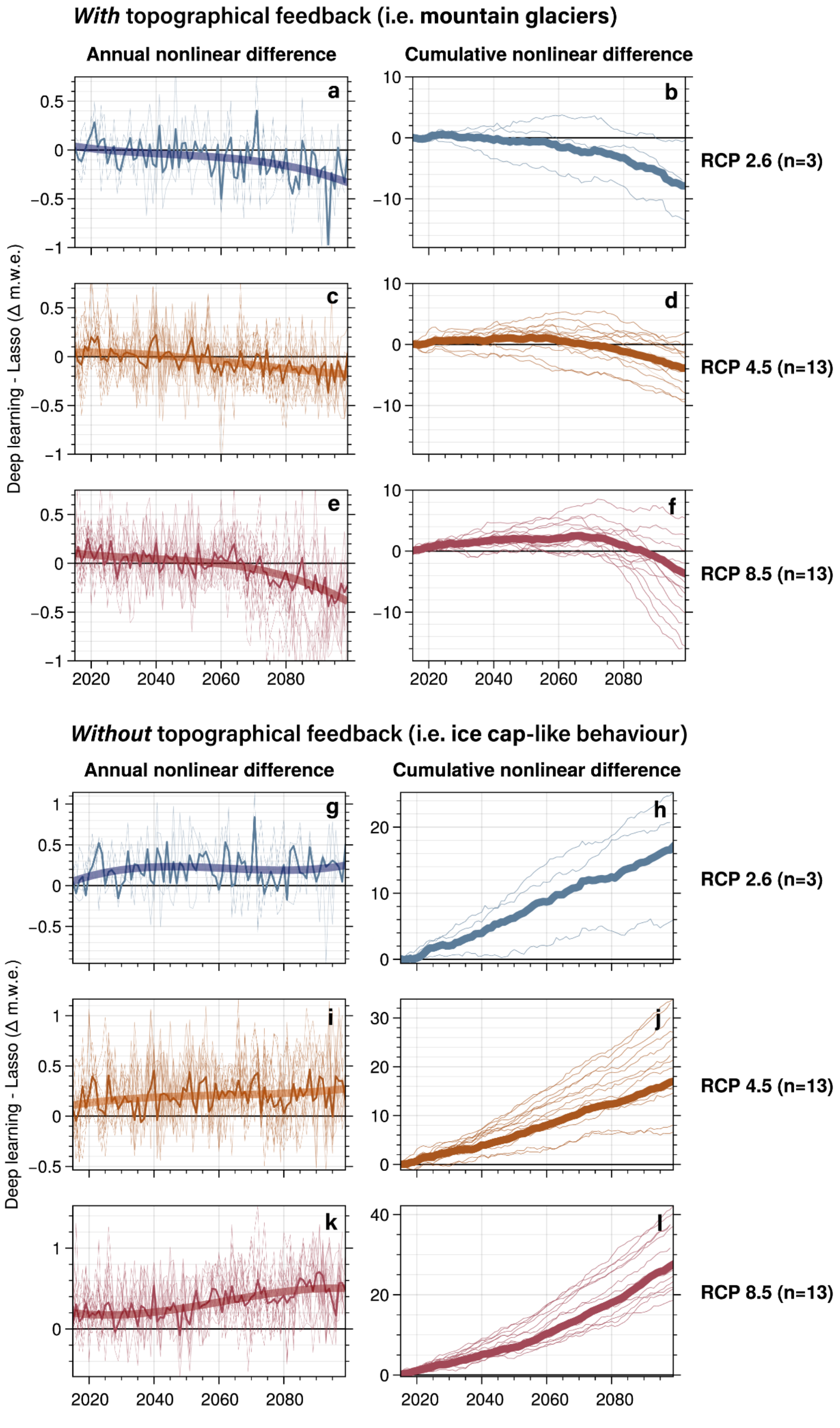

Fig. S5. Effects of deep learning nonlinearities on mass balance projections. Comparison between nonlinear (deep learning) and linear (Lasso) MB projections for the French Alps dataset with topographical feedback (a-f), and from a synthetic experiment without topographical

feedback, keeping glacier geometry constant (g-l). The constant glacier centroid where the climate data is computed serves to simulate climate conditions undergone by ice caps.

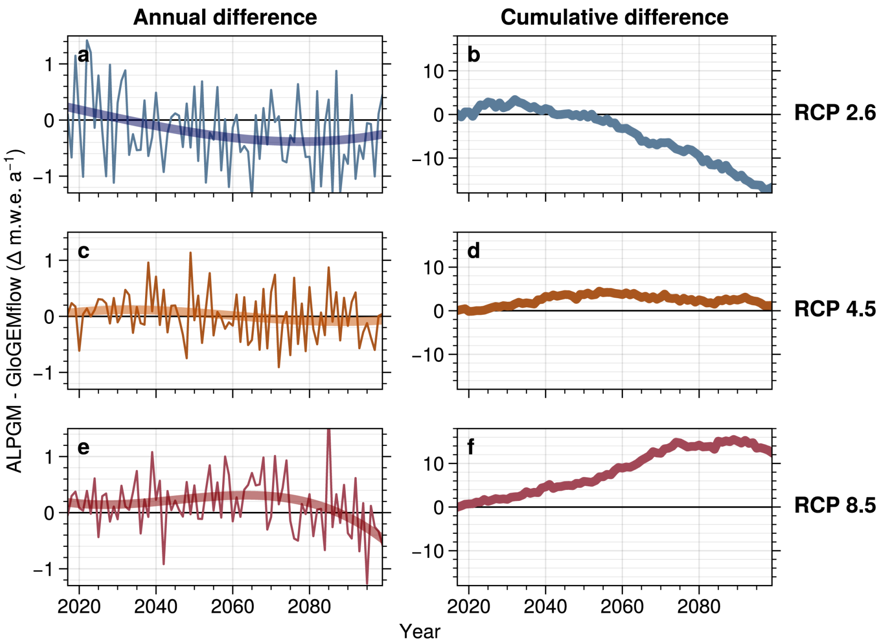

Fig. S6. Effects of deep learning nonlinearities compared to a temperature-index mass balance model. Difference in average annual glacier-wide MB between the ALPGM (nonlinear MB model, this study) and GloGEMflow (linear MB model) glacier evolution models for RCPs 2.6 (**a-b**), 4.5 (**c-d**) and 8.5 (**e-f**). MB data from GloGEMflow have been adjusted by adding a bias computed between ALPGM and GloGEMflow for the 2003-2015 period to improve comparability (see Materials and Methods).

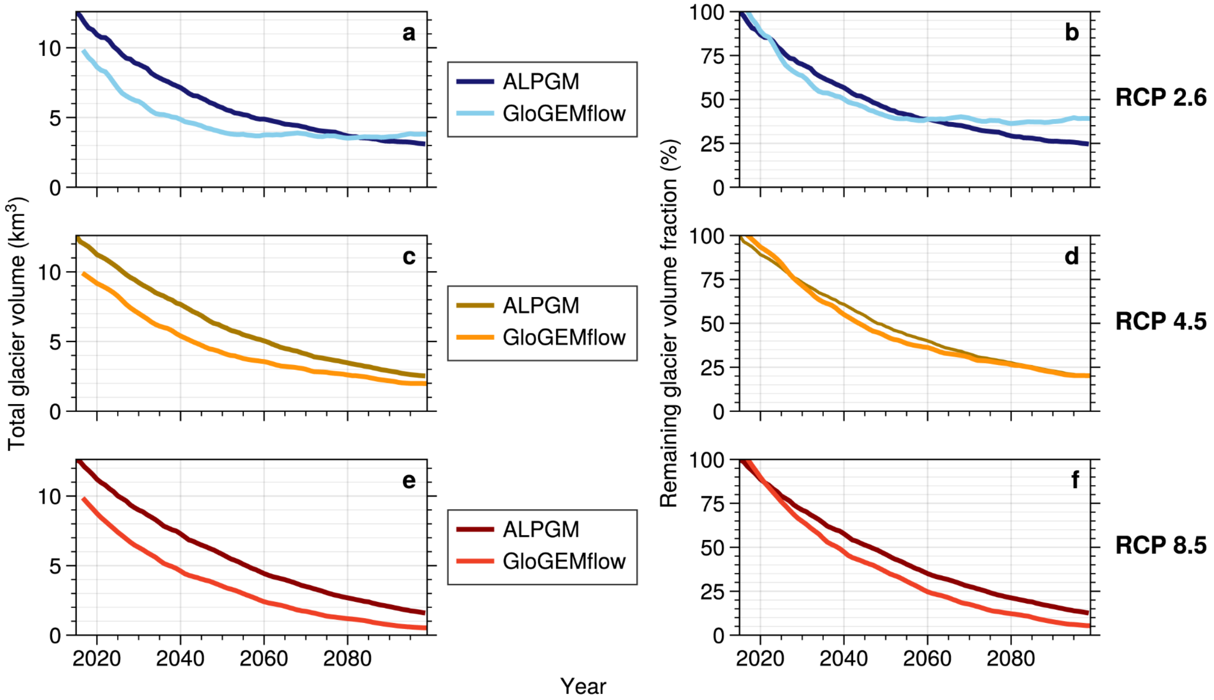

Fig. S7. Comparison of projected glacier volumes between ALPGM (this study) and GloGEMflow (temperature-index model). The initial ice thickness estimates are different (a, c, e), with the relative ice volume change displayed in b, c, f. These changes show similar trends to the ones observed in Fig. 5.

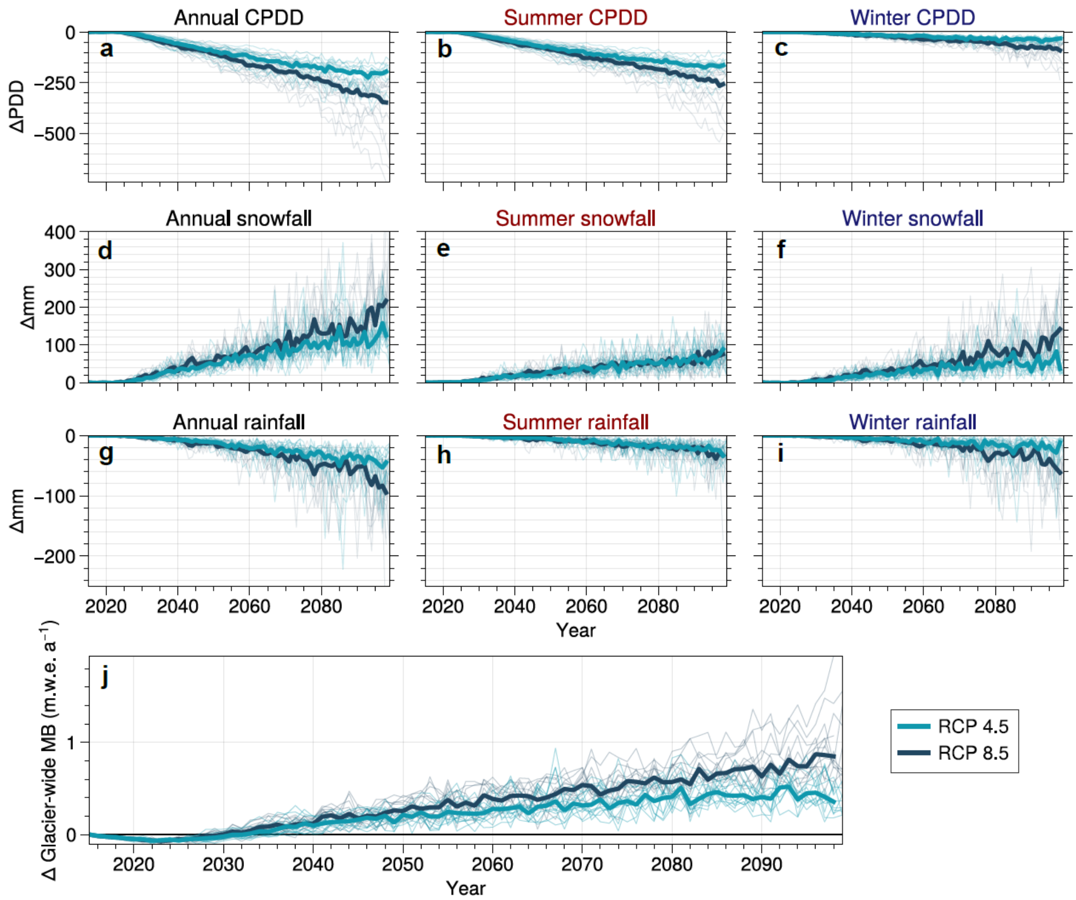

Fig. S8. Glacier retreat effects on the climate signal of glaciers. Computed as the difference between model runs with glacier dynamics and model runs without glacier dynamics (i.e. static glaciers). Glaciers adjusting their geometry by shrinking to higher altitudes modify their received climate signal (**a-i**). These changes in the received climate help mitigate their mass losses, in an effort to reach equilibrium with the present climate (**j**).

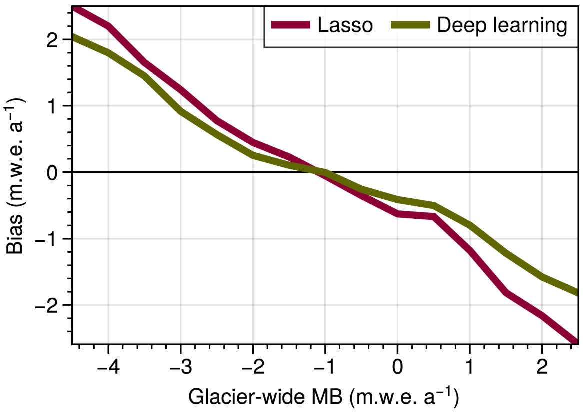

Fig. S9. Glacier-wide MB bias for the Lasso and deep learning models. Average annual glacier-wide MB bias for the Lasso and deep learning MB models. Values computed using LSYGO cross-validation, based on data for the 1967-2015 period.

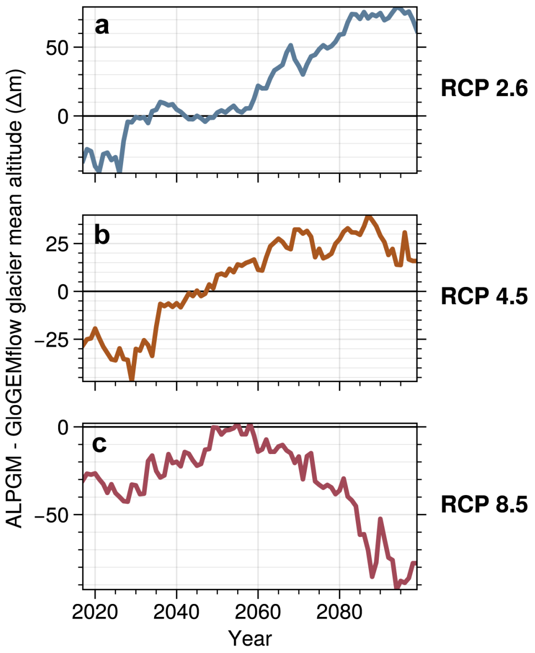

Fig. S10. Mean glacier altitude difference between ALPGM and GloGEMflow. Mean annual glacier altitude difference between ALPGM (this study) and GloGEMflow for **(a)** RCP 2.6, **(b)** RCP 4.5 and **(c)** RCP 8.5.

Supplementary tables

ADAMONT climate projections member	Available RCPs
CLMcom-CCLM4-8-17_CNRM-CERFACS-CNRM-CM5	RCP 4.5<br>RCP 8.5
CLMcom-CCLM4-8-17_ICHEC-EC-EARTH	RCP 4.5<br>RCP 8.5
CLMcom-CCLM4-8-17_MOHC-HadGEM2-ES	RCP 4.5<br>RCP 8.5
CLMcom-CCLM4-8-17_MPI-M-MPI-ESM-LR	RCP 4.5<br>RCP 8.5
DMI-HIRHAM5_NCC-NorESM1-M	RCP 4.5<br>RCP 8.5
IPSL-INERIS-WRF331F_IPSL-IPSL-CM5A-MR	RCP 4.5<br>RCP 8.5
KNMI-RACMO22E_MOHC-HadGEM2-ES	RCP 2.6<br>RCP 4.5<br>RCP 8.5
MPI-CSC-REMO2009_MPI-M-MPI-ESM-LR	RCP 2.6<br>RCP 4.5<br>RCP 8.5
SMHI-RCA4_CNRM-CERFACS-CNRM-CM5	RCP 4.5<br>RCP 8.5
SMHI-RCA4_ICHEC-EC-EARTH	RCP 2.6<br>RCP 4.5<br>RCP 8.5
SMHI-RCA4_IPSL-IPSL-CM5A-MR	RCP 4.5<br>RCP 8.5
SMHI-RCA4_MOHC-HadGEM2-ES	RCP 4.5<br>RCP 8.5
SMHI-RCA4_MPI-M-MPI-ESM-LR	RCP 4.5<br>RCP 8.5

Table S1. List of the 29 climate members used to force the glacier evolution model. Climate members are composed by a combination of GCM-RCM-RCP. Since only three members include RCP 2.6, separate analyses have been performed using only these members in order to have comparable climate variabilities.

	**This study: ALPGM**	**Zekollari et al. (2019): GloGEMflow**
**MB component**	Deep learning	Accumulation and temperature-index melt model. The MB component is the same one as in GloGEM (Huss and Hock, 2015)
**Glacier dynamics component**	Glacier-specific parametrizations for glaciers > 0.5 km^2 (Δh method). Equal loss distributed over all glacier altitudes for glaciers < 0.5 km^2, representing non-dynamic downwasting.	Ice flow dynamics based on shallow ice approximation along the flowline (for glaciers > 1 km) and three generalized retreat parameterizations based on Δh method (for glaciers < 1 km)
**MB calibration data**	1048 values of annual glacier-wide MB from glaciological observations and remote sensing estimates for the French Alps	Calibration based on geodetic mass balances, covering 38% of all glaciers in the European Alps (mainly in Switzerland), corresponding to about 60% of the total glacier area.
**Climate projection forcing**	High-resolution (300 m altitude bands divided by massifs) mountain-adjusted climate forcings with 13 GCM-RCM member combinations	EURO-CORDEX ensemble at 0.11° resolution
**Glacier ice thickness**	Farinotti et al. (2019) + field measurements	Huss and Farinotti (2012)

Table S2. Comparison of glacier evolution models characteristics. Differences between the glacier model used in this study (ALPGM) *vs* the glacier model used in Zekollari et al., 2019 (GloGEMflow).

References

1. Smit, M., Amelung, S. B. & Meijering, J. J. Exploring intergenerational attitudes toward glacier retreat in the Chamonix Mont-Blanc Valley, France. (2019).
2. Mourey, J. & Ravanel, L. Evolution of Access Routes to High Mountain Refuges of the Mer de Glace Basin (Mont Blanc Massif, France): An Example of Adapting to Climate Change Effects in the Alpine High Mountains. *Rev. Géographie Alp.* (2017) doi:10.4000/rga.3790.
3. Bourdeau, P. Examining innovation in the Alps at the local scale: A region on the move, the "Pays des Ecrins". *Rev. Géographie Alp.* (2009) doi:10.4000/rga.797.
4. Schut, P.-O. Sport as a Major Player in the Development of Tourism: The History of Mountaineering in the Pelvoux Massif, France, from 1861 to 1914. *Int. J. Hist. Sport* **30**, 1329–1350 (2013).
5. Mackintosh, A. N., Anderson, B. M. & Pierrehumbert, R. T. Reconstructing Climate from Glaciers. *Annu. Rev. Earth Planet. Sci.* **45**, 649–680 (2017).
6. Bolibar, J. *et al.* Deep learning applied to glacier evolution modelling. *The Cryosphere* **14**, 565–584 (2020).
7. Thibert, E., Dkengne Sielenou, P., Vionnet, V., Eckert, N. & Vincent, C. Causes of Glacier Melt Extremes in the Alps Since 1949. *Geophys. Res. Lett.* **45**, 817–825 (2018).
8. Zemp, M. *et al.* Global glacier mass changes and their contributions to sea-level rise from 1961 to 2016. *Nature* **568**, 382–386 (2019).
